# Point-of-care ultrasonography in nephrology: a cross-sectional national survey among Brazilian nephrologists

**DOI:** 10.1590/2175-8239-JBN-2020-0023

**Published:** 2020-10-05

**Authors:** Marcus G. Bastos, Ana Luisa Vieira, Marcelo Mazza do Nascimento, Elvino Barros, José Muniz Pazeli, Gianna Mastroianni Kirsztajn

**Affiliations:** 1 Universidade Federal de Juiz de Fora, Juiz de Fora, MG, Brasil.; 2 Faculdade de Medicina de Barbacena, Barbacena, MG, Brasil.; 3 Universidade Federal do Paraná, Curitiba, PR, Brasil.; 4 Universidade Federal do Rio Grande do Sul, Faculdade de Medicina, Departamento de Medicina Interna, Porto Alegre, RS, Brasil.; 5 Universidade Federal de São Paulo, Escola Paulista de Medicina, Departamento de Medicina, São Paulo, SP, Brasil.

**Keywords:** Ultrasonography, Nephrology, Ultrasonics, Education, Mentoring, Research, Ultrassonografia, Nefrologia, Ultrassom, Educação, Tutoria, Pesquisa.

## Abstract

**Introduction:**

Point-of-care ultrasonography (US) (POCUS) has been used in several specialties, particularly in medical emergency. Despite the confirmation of its numerous benefits, the use of POCUS is still timid in nephrology. In the present study, we aim to investigate the use of POCUS by Brazilian nephrologists.

**Methods:**

A survey carried out among the members of the Brazilian Society of Nephrology, through institutional e-mail, using the SurveyMonkey platform. We included 12 self-administered questions, which answers were given anonymously.

**Results:**

It was evident that the majority (64%) of the participants did not have the opportunity to practice US during their nephrological training in their residency, specialization, or even in internships; those with experience with US use the method mainly for implanting central vascular accesses (68%), performing a renal biopsy (58%) and evaluating renal morphology (50%); and the main barriers for nephrologists who do not yet use US are the high price of US machines (26%) and the lack of time to learn about US (23%). Also, POCUS use for examinations of other organs, such as the lung (31%) and heart (18%), which are fundamental in the cardiovascular and volume assessment of patients with kidney diseases, is even more limited. However, 95% of the participants expressed an interest in learning POCUS for use in their medical practice.

**Conclusion:**

Most of the Brazilian nephrologists interviewed were not trained in US; however, almost all of the research participants expressed an interest in learning to use POCUS in nephrological practice.

## Resumo

**Introdução:**

A ultrassonografia (US) pointof-care (POCUS) tem sido utilizada emvárias especialidades, particularmente na urgência médica. Apesar da constatação de seus numerosos benefícios, a utilização da POCUS ainda é tímida na nefrologia. No presente estudo, objetivamos fazer um levantamento sobre a utilização da POCUS pelos nefrologistas brasileiros.

**Métodos:**

Levantamento realizado entre os sócios da Sociedade Brasileira de Nefrologia, por meio do e-mail institucional, utilizando a plataforma SurveyMonkey. Foram incluídas 12 perguntas autoadministradas, cujas respostas se deram de forma anônima.

**Resultados:**

Ficou evidente que a maioria (64%) dos participantes não teve oportunidade de praticar a US durante sua formação nefrológica na residência, especialização ou mesmo em estágios; que aqueles com experiência com a US usam o método, principalmente, para implantação de acesso vascular central (68%), realização de biópsia renal (58%) e avaliação da morfologia renal (50%); e que as principais barreiras para os nefrologistas que ainda não utilizam a US são o preço elevado das máquinas de US (26%) e a falta de tempo para aprender sobre US (23%). Além disso, o uso da POCUS para exames de outros órgãos, como pulmão (31%) e coração (18%), fundamentais na avaliação cardiovascular e volêmica dos pacientes com doenças renais, ainda é mais limitado. Porém, 95% dos participantes expressaram interesse em aprender a POCUS para aplicação na sua prática médica.

**Conclusão:**

A maioria dos nefrologistas brasileiros entrevistados não foi treinada em US, contudo, a quase totalidade dos participantes da pesquisa manifestou interesse em aprender a utilizar a POCUS na prática nefrológica.

## Introduction

A quality physical examination (PE) is essential in the diagnostic process. Although it is indisputable, it is important to recognize that traditional PE has incorporated a few new technologies, with the stethoscope in 1816 being the most important one.^1^

However, traditional PE does not enable us to “look under the skin” of the patient. This requires imaging techniques. Among imaging methods, ultrasound (US) has gained wide acceptance and use, particularly among non-radiologists, for not using ionizing radiation, allowing for dynamic studies, not being invasive, and being used to guide procedures. Besides, excellent portability (today units can fit in the in the palm of your hand), the development of US applications that work on **smartphone**, and the gradual decrease in cost make US a method with enormous potential for incorporation into daily clinical practice.^2^

US has evolved considerably in the last decades as an image examination modality and, worldwide, 25% of the medical images generated are ultrasonographic.^3^ Guidelines have encouraged the incorporation of US as an additional propaedeutic method, in the guidance of procedures and the training of residents of different medical specialties.^4^^-^ 6

Alterations found in the physical examination can be further investigated, at the bedside, with US allowing expanding the clinical information and direct diagnostic and therapeutic interventions. Therefore, it is necessary to stimulate this new technology in medical training, especially in nephrology. Nephrology residents and interns must be exposed not only to the interpretation of US images generated by specialists but also to handle the US equipment to become competent to obtain and interpret US images when performing the PE.

We hypothesize that most nephrologists in training or practice have limited knowledge about “Point-of-Care” (POCUS), but are interested in acquiring training to include this propaedeutic in evaluating patients and the performance of procedures.

This study aims to survey the practice of US among physicians in training or the practice of nephrology in Brazil.

## Methods

We conducted a national cross-sectional online study on the use of US by nephrologists in collaboration with the Brazilian Society of Nephrology (SBN). We then sent a structured questionnaire using the SurveyMonkey platform by institutional e-mail to nephrologists in the database of active members of the SBN. Each electronic link was restricted in order to enable the participant to respond only once. E-mails were sent five times from March to August 2019, as a strategy to increase the number of respondents. The Research Ethics Committee of the Barbacena School of Medicine approved the study (CAAE: 02789818.0.0000.8307).

We included 12 self-administered questions, which answers were given anonymously, and the participants did not receive any payment or other benefits to answer the questionnaire. We designed the questions to identify potential barriers/challenges and prerequisites for the use of US in clinical practice, ensuring response confidentiality. The questionnaire covered demographic data, conditions/locations of nephrology practices, years of practice in nephrology, previous experience in the use of urinary tract US and other organs/systems (heart, lung, inferior vena cava, others), opportunity to use US in the period of professional training and current availability of ultrasound machines in the workplace. The final question referred to the respondent’s interest in learning about US to assess patients and perform procedures.

The data obtained are presented as frequency using descriptive statistics.

## Results

After five rounds of sending emails to the 3,500 contacts on the SBN mailing list, we recorded 3,425 openings of the questionnaire, with 609 clicks and 517 respondents who signed the Free and Informed Consent Form, making a total rate of 15% of participants. The survey completion rate was 100%, and the average questionnaire response time was two minutes and five seconds.

Sections of the survey revealed results in several aspects. Regarding demographic data, of the total nephrologists participating in the study, 55.5% were male, with the highest percentage of respondents (40%) corresponding to the age group between 31 and 40 years of age, and 75% with 11 or more years in the profession. Seventy-one percent practiced nephrology in public and private institutions, 78% of which have an US machine, which could be used by the vast majority (72%) of nephrologists in case of need (Table 1).

**Table 1:** Demographic data and availability of ultrasound machines included in the questionnaire sent to nephrologists who are members of the Brazilian Society of Nephrology

Sex, n (%)	
Male	287 (56)
Female	230 (44)
Participant's age, n (%)	
20-30 years	24 (5)
31-40 years	208 (40)
41-50 years	134 (26)
>50 years	151 (29)
Years after graduation, n (%)	
1 to 2 years	10 (2)
3 to 5 years	29 (6)
6 to 10 years	92 (18)
11 to 20 years	192 (37)
>20 years	194 (38)
After training, you practice nephrology in what institution: n (%)	
Public	67 (13)
Private	85 (16)
Public and private	367 (71)
Is there an ultrasound machine available in the place where you work today? N (%)	
Yes	403 (78)
No	114 (22)
If you answered YES to the previous question, and in case you need, can you use it? n (%)	
Yes	371 (72)
No	50 (10)
Do not know	96 (19)

**Figure 1:**
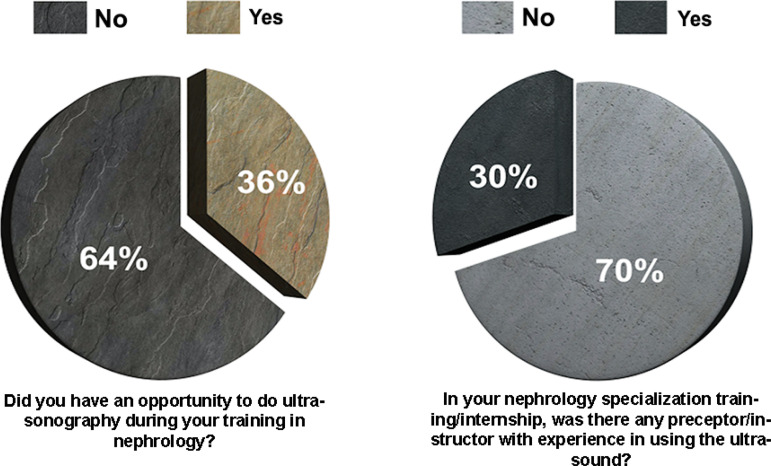
Learning ultrasound during nephrological training.

Concerning the learning about US use during training in nephrology, 64% answered that they had no opportunity to use the US. The majority (70%) answered that there was no instructor/preceptor with experience performing the US when training in nephrology (Figure 1).

As for the procedures with the use of US performed by nephrologists, central vascular access (68%), renal biopsy (58%) and renal evaluation (50%) constitute the procedures that nephrologists who already use POCUS (50% respondents) perform more frequently. Inferior vena cava (32%), lungs (31%), and heart (18%) USG are still relatively underutilized (Table 2).

**Table 2:** Respondents' clinical use of ultrasonography in their practice of nephrology

Clinical utilization	Point of care ultrasound N (%)
Central venous access	353 (68.28)
Renal biopsy	300 (58.03)
Kidney assessment	260 (50.29)
Urinary bladder assessment	201 (38.88)
Inferior vena cava assessment	164 (31.72)
Pulmonary ultrasound	161 (31,14)
Heart assessment	93 (17.99)
Abdominal aorta assessment	64 (12.38)
Carotid artery assessment	48 (9.28)
Not informed	92 (18.79)

**Figure 2:**
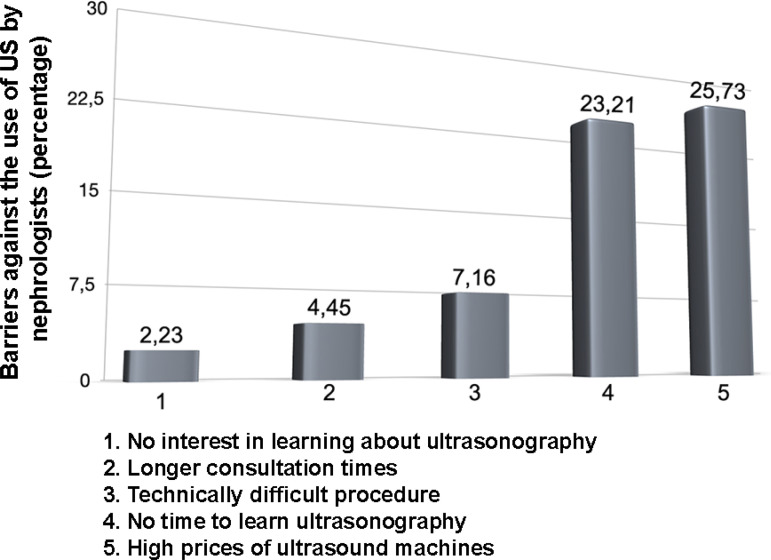
Barriers against the use of ultrasound by the nephrologist.

Concerning the use of US by nephrologists, the barriers mentioned were the high price of US machines (26%); lack of time to learn about US (23%); perception of being a difficult procedure (7%); increased consultation time (4.5%) and lack of interest in learning US (2%) (Figure 2). When asked about their interest in learning US to assess their patients and perform procedures, 95% of participants responded affirmatively.

The participants also made several suggestions and had many comments on the use of US by nephrologists, among which we highlight some testimonies: 1. “Disseminate the importance of US in nephrological practice and encourage the mandatory teaching of this modality in Nephrology Medical Residency programs”; 2. “The SBN could promote traveling training/refresher courses in US to its members”; 3. “Creation of specific courses or during events, such as a pre-congress course, including assessment of adults and children through US“; 4. “US panels and discussions in large conventions”; 5. I have been practicing POCUS for two years, and I believe it is essential not only for nephrologists but also for any physicians. Paradigm shifts as an extension of the patient’s clinical examination. It is absurd that we still do not have a portable US in all ICUs, emergency wards of clinics and nephrology clinics. The practice of ultrasound is pleasurable and opens new paths. It is the doctor’s third eye and far surpasses the vision and reasoning compared to the most expert propaedeutic. We are 20 years behind Europe and the USA. Let us go after it. and 6. “Extremely important in current practice. I think that not only for vascular accesses, but we need US courses for lung, cava, and to estimate blood volume more safely”.

## Discussion

Non-specialist physicians have used POCUS to obtain, interpret, and use US images when performing a physical examination of the patient, as a diagnostic instrument of extraordinary value in various medical specialties. As far as we know, this is the first study of a cross-sectional survey related to the use of POCUS in multisystem diagnoses among Brazilian nephrologists. The study shows that the majority of participants were not trained in US; however, almost all respondents expressed an interest in learning to use POCUS in nephrological practice.

Results found in several medical specialties have demonstrated the beneficial use of POCUS concerning clinical outcomes, reduced failures, and complications during invasive procedures. US can aid in the diagnosis and management of acute disease processes.^7^^-^ 11 However, in nephrology, particularly in Brazil, POCUS's integration in clinical evaluation is not widespread. In the study presented, it was clear that most of the participating nephrologists are not yet trained in POCUS, even when working in institutions where US equipment is available. Among those who have already perform POCUS, the US is often used to guide invasive procedures and perform a renal assessment. It was also clear that the main barriers to incorporating POCUS in the nephrological assessment are the price of US machines and the lack of time to learn to use it. However, 95% of the respondents are interested in learning how to use the US and made suggestions for more insertion of POCUS in local or national nephrology congress, provide pre-congress courses and even itinerant training courses.

An interesting finding was the high percentage of US machine availability in institutions, public and private, with a potential for use by nephrologists in case of need. Although it was not specifically asked, it is not difficult to imagine that such availability of US machines occurs, almost exclusively, in dialysis units and infirmary in a hospital environment, and not in clinics of renal replacement therapy. However, in order to perform US, it is necessary to know how to operate the equipment, either in performing procedures guided by the method (renal biopsy, central venous access) as well as in obtaining images in the nephrologist’s operating scenery.

As mentioned, one of the barriers to the insertion of US in the nephrological evaluation is the lack of time for the nephrologist. However, it is necessary to emphasize that, at POCUS, the objective is “yes” or “no” responses to focused questions, such as, for example, in the dyspneic patient who did not attend the last two hemodialysis sessions, is there pulmonary congestion? Yes or no? Therefore, short-term training may be sufficient to enable the nephrologist to answer such questions.

Recent publications suggest that groups of resident physicians acquire skills for the generation and interpretation of US images after short training (3 to 16 hours) at POCUS, structured with theoretical classes and practical sessions.^12^^-^ 14 So far, the number of US examinations required to enable the nephrologist to perform the POCUS in nephrology has not been established, but, as with the acquisition of other medical skills, the learning curve is directly related to the number of USG performed and the frequency of US use.

One aspect that has hindered POCUS in nephrology is the lack of training for residents (or other graduate students) due to their preceptors/instructors' inexperience in performing US. Such difficulty could be easily overcome, as POCUS training requires only a physician familiar with the procedure and a simpler US machine, portable or even ultraportable, whose images are generated on **smartphones** or **tablets**. As it is a relatively new skill, but useful in practically all medical specialties,^2^ finding properly trained preceptors to teach POCUS is still an significant limitation. Currently, the interpretation of US images generated by specialists is part of the list of knowledge to be acquired in the training of nephrologists; however, residents are not required to perform the procedure.

It is noteworthy that, among nephrologists who already perform US, the POCUS is more often used to guide procedures (renal biopsy and central venous access) and limited to the study of the kidneys, as suggested until recently.^2^^,^ 15 ^,^ 16 However, because the kidneys are multifunctional organs that, when dysfunctional, determine acute or chronic repercussions in various sectors of the economy, POCUS in nephrology should transcend the urinary tract's limits. 14 ^,^ 24 ^,^ 25

In this context, pulmonary POCUS has been gaining special attention. For example, Zoccali et al^17^ observed that asymptomatic pulmonary congestion detected through the pulmonary US in patients undergoing hemodialysis is strong and independent predictor of mortality and cardiac events. Likewise, focused echocardiography (ECF) is another skill nephrologists must incorporate that can even be performed using ultraportable US.^18^ ECF enables one to quickly detect imminent death conditions, such as cardiac tamponade, as well as situations of hypervolemia and hypovolemia, ventricular hypertrophy and diastolic and systolic dysfunctions of the left ventricle, common in patients at different stages of chronic kidney disease.^19^^-^ 24

It is worth mentioning that, to date, there are no international studies on the use of US in nephrology, which makes it difficult to assess our findings comparatively. However, the perception of the importance of POCUS in the multisystem diagnosis in nephrology could be synthesized in the words of O’Neill and Ross^25^:

"We should not continue to practice nephrology and train future nephrologists in the same way that we did 25 years ago. Point-of-care ultrasound is rapidly being incorporated into medical practice, and not including it in the training of young nephrologists will leave us behind, much like what happened to physicians who never adopted the stethoscope."

## Study Limitations

The study has limitations, particularly about respondents' rate, despite the repeated messages sent by institutional e-mails. Although 100% of the participants answered all questions, the response rate limits the results' generalization. Self-selection bias may have occurred in respondents with a greater interest in learning POCUS, and perceived its importance, restricting the generalization of results. We were also unable to identify the different regions of Brazil from which the participants came, which may have affected the percentage of responses regarding the availability of US machines, previously trained preceptors, and the use of POCUS. It is important to note that the survey has not been formally validated. Because it involves retrospective self-reporting, some respondents may have responded differently about how they use POCUS.

## Conclusion

The study with participating Brazilian nephrologists shows that a still relatively small percentage of respondents have already used POCUS in nephrological practice. Among those who already use US, the most frequent use is to guide invasive procedures (central venous access and renal biopsy) and evaluate the kidneys. The non-invasive nature of the method, portability, and the more affordable prices of US equipment, together with the desire to obtain POCUS training that the absolute majority of respondents expressed, enable us to foresee that US will be quickly incorporated into national nephrology practice.
